# Extrapolation of affective norms using transformer-based neural networks and its application to experimental stimuli selection

**DOI:** 10.3758/s13428-023-02212-3

**Published:** 2023-09-25

**Authors:** Hubert Plisiecki, Adam Sobieszek

**Affiliations:** 1 Institute of Psychology, Polish Academy of Sciences, SWPS University of Warsaw, Warsaw, Poland; 2 Faculty of Psychology, University of Warsaw, Warsaw, Poland

**Keywords:** Affective norms, Transformer-based neural network, Semantic extrapolation, Emotional norms extrapolation, Experimental stimuli selection, Valence and arousal

## Abstract

Data on the emotionality of words is important for the selection of experimental stimuli and sentiment analysis on large bodies of text. While norms for valence and arousal have been thoroughly collected in English, most languages do not have access to such large datasets. Moreover, theoretical developments lead to new dimensions being proposed, the norms for which are only partially available. In this paper, we propose a transformer-based neural network architecture for semantic and emotional norms extrapolation that predicts a whole ensemble of norms at once while achieving state-of-the-art correlations with human judgements on each. We improve on the previous approaches with regards to the correlations with human judgments by Δ*r* = 0.1 on average. We precisely discuss the limitations of norm extrapolation as a whole, with a special focus on the introduced model. Further, we propose a unique practical application of our model by proposing a method of stimuli selection which performs unsupervised control by picking words that match in their semantic content. As the proposed model can easily be applied to different languages, we provide norm extrapolations for English, Polish, Dutch, German, French, and Spanish. To aid researchers, we also provide access to the extrapolation networks through an accessible web application.

**Supplementary Information:**

The online version contains supplementary material available at 10.3758/s13428-023-02212-3.

Affective norms of words have various applications across psychology, linguistics, and machine learning. Their importance is evidenced by the large number of use cases they enjoy. They have been used to select stimuli for experiments in social and affective psychology to investigate behavior (Crossfield and Damian, 2021), to study clinical populations (Williamson et al., 1991; Sloan et al., 2001), and as correlates of brain activity (Citron, 2012; Imbir et al., 2022; Kanske & Kotz, 2007; Yao et al., 2016). Together with norms of semantic dimensions they serve as tools for the study of lexical semantics, concerned with how concepts may be represented in the brain (Binder et al., 2016). Recently, semantic and affective norms have seen a surge in popularity with the growing interest in machine learning, where they have been used to train automatic classifiers of, for example, the sentiment expressed in a given piece of text (Nielsen, 2011). All such uses rely on databases of word – norm pairs, where norms are calculated based on human ratings of the word on a particular dimension of interest (e.g., how positive, or negative a given word is, a technique dating back to the work of Osgood et al., 1957). To this end, measurement scales for various lexical affective constructs have been developed, starting with the simple Likert scale, and continuing with the popular self-assessment manikin of Bradley and Lang (1994).

The two most popular approaches in creating emotional norms include either rating words on emotional dimensions or their association with discrete emotion categories. The most popular of these dimensions in the first approach include valence, arousal, and dominance (Bradley & Lang, 1999; Imbir, 2015; Sianipar et al., 2016; Söderholm et al., 2013; Stadthagen-Gonzalez et al., 2017; Verheyen et al., 2020; Warriner et al., 2013; Yao et al., 2017) and to a lesser extent other dimensions which may modulate emotional processing, such as concreteness, age of acquisition, subjective significance, or origin of emotional load (Brysbaert et al., 2014a, b; Imbir 2016; Kuperman et al., 2012). As for discrete emotional categories, norms are usually concerned with subsets of the six basic emotions: fear, anger, joy, sadness, disgust, and surprise (Mohammad, 2018; Stevenson et al. 2007). The affective norms have been published for many languages other than English (Bradley & Lang, 1999): French (Syssau et al., 2021), German (Võ et al., 2009), Spanish (Redondo et al., 2007), Dutch (Moors et al., 2013), Polish (Imbir, 2016), Turkish (Kapucu et al., 2021), Italian (Montefinese et al., 2014), Portuguese (Soares et al., 2012), Greek (Vaiouli et al., 2023), and Chinese (Yao et al., 2017).

Some applications of affective norms, such as complex experimental designs, demand very large datasets, the creation of which can be prohibitively expensive. This demand has been partially satisfied for English words, with the expansion of the classic Affective Norms for English Words database (ANEW; Bradley & Lang, 1999) from 1035 to 13,915 by Warriner et al. (2013). However, such large dataset expansions are still unavailable for many languages. Moreover, even in English, the existing norms may not be enough for certain types of studies, which could require norms for *all* English words. Interesting examples of such include analyses of large-scale trends and shifts in the use of language across thousands – if not millions of texts (Kim & Klinger, 2019), where an accurate assessment may require a rating for each word to avoid bias (Snefjella & Blank, 2020). It is in this context that *lexical norm extrapolation* techniques start to be developed, as they allow researchers to use existing norms to expand the database lexicon by predicting the norms of previously unrated words.

## Affective norms extrapolation

How does one go about determining the emotionality of words without any human judgment information? A first intuition may be to say that the emotional load of a word can be approximated with the emotional load of another, *similar*, word for which we possess affective norms. This indeed turns out to be the basis for most published norm extrapolation techniques, the difference being mostly in the level of sophistication with which the similarity metrics are defined and the introduction of ways to decrease noise by averaging across many similar words. A popular source of similarity metrics comes from the linguistic distributional hypothesis, which states that words that occur in similar contexts tend to have similar meanings (Boleda, 2020). Thus, the dominant approach in affective norms extrapolation is usually to average a norm of interest across a word's k-nearest neighbors based on a co-occurrence metric, which the review by Mandera et al. (2015) deemed the most effective method as of 2015. An early example of such an approach includes Bestgen and Vincze’s (2012) use of latent semantic analysis to derive similarities between words, where co-occurrence is calculated from paragraphs of large language corpora. While varying the number of neighbors to average across, they found the highest correlations between human ratings and their estimates to be *r* = 0.71 for valence, *r* = 0.56 for arousal, and *r* = 0.60 for dominance.

More recent approaches used in machine learning for sentiment analysis employ similarity metrics based on distances in a vector space, where words are represented as points (e.g., Munikar et al., 2019). These spaces, called *word embeddings*, are intended to be lower-dimensional representations of the relationships between the words in language corpora and are created in various ways, which include dimensionality-reduction techniques on the co-occurrence matrix (e.g., multidimensional scaling) and the use of neural networks (e.g., in word2vec; Mikolov et al., 2013).

A different approach has been employed (to great effect) by Vankrunkelsven et al. (2015). Their method involves using a vast dataset of word associations (De Deyne et al., 2013), which are based on 70,000 participants reporting their three associations with one of 12,000 cue words. Since free associations are often based on semantic relationships with the cue word, these data can be used to construct a great similarity metric. Vankrunkelsven et al. used multi-dimensional scaling to construct word embeddings based on this data and achieved correlations of *r* = 0.89, *r* = 0.76, *r* = 0.77, *r* = 0.67, and *r* = 0.81, for valence, arousal, dominance, age of acquisition, and concreteness, respectively. As semantic norm extrapolation is most useful for languages where access to such data is limited, the need to collect vast word association data to perform norm extrapolation seems, while elegant, to be of limited practical utility. Still, a comparison by Vankrunkelsven et al. (2018) of their association-based method with previous methods based on co-occurrence shows that their method achieved state-of-the-art results for the time.

A challenge for all such extrapolation methods, recently presented by Snefjella and Blank (2020), posits that researchers may, however, be overestimating the accuracy of their methods of norm extrapolation by relying on cross-validation to evaluate performance. This is because the words that are missing from the norm databases (in which we are ultimately interested in extrapolation) are not missing at random from all possible words. They suggest considering norm extrapolation as missing data imputation.

## Advances in neural network-based language models

In recent years, the field of computational linguistics has been taken by storm with rapid developments in neural network-based models. especially large language models, one of the most notable ones is called GPT-3 and was trained on a corpus of text comprising nearly 500 billion words (Brown et al., 2020). The performance of this model varies, as it has been shown to perform with near human ability on many high-level tasks like imitating an author or waxing philosophical while failing at several very simple tasks like multiplying large numbers (Elkins & Chun, 2020; Sobieszek & Price, 2022). It is, however, worth stressing that on many occasions its performance is indistinguishable from that of a human and that this high performance is not merely the product of the sheer size of the training dataset. GPT-3, as well as its predecessors GPT-2 and GPT, utilize a specific machine learning architecture called *attention*, which allows them to attend to many distant words at once, thus being able to grasp complicated contextual information when analyzing text (Brown et al., 2020).

These developments lie in contrast to the previous attempts at text classification, translation and production in natural language processing, as previous models were very limited in their scope when it comes to attending to distant words. The earliest approaches utilized the already-mentioned word embeddings, which were heavily dependent on closely co-occurring words, and thus were unable to capture relations between distant signs (Almeida & Xexéo, 2019). The situation improved with the rise of recurrent neural networks such as LSTMs (Long Short-term memory modules) which tried to retain significant textual information as they gradually advanced through the lines of text (Yu et al., 2019). Unfortunately, these approaches were plagued by the problem of vanishing gradients, resulting in the retained information being lost over time as the activation progressed through the network (Hochreiter, 1998). They were therefore short-sighted. Afterwards came convolutional networks (CCN), which compressed contextual information using sliding windows, thus capturing their contents (Yin et al., 2017). These had a different flaw, however, as to capture complex contextual information one had to apply very large sliding windows (buffers for word compression), and many of them – which was incredibly costly in terms of computational power (Vaswani et al., 2017).

Finally, the concept of “attention” was introduced, which is in simple terms the process of weighing the inputs to a neural layer with the use of trainable weights. This method was first applied in recurrent neural network-based sequence-to-sequence models, which iteratively passed the generated sequence through a generator module to obtain the next word, appended the word to the generated sequence and repeated the process until the whole sequence of interest was generated. The real breakthrough, however, came when the recurrent network architecture was replaced by attention. This feat, achieved by Vaswani et al. (2017), morphed into a family of models called transformers, some among which are BERT, RoBERTa, and XLM (Devlin et al., 2018; Conneau & Lample, 2019; Liu et al., 2019).

Transformer models consist of two modules, an encoder, and a decoder. As this architecture was primarily designed for the task of language translation, we will use the task of language translation as a reference when explaining its mechanism. In simple terms, a sentence in the 1st language is given to the encoder, which transforms it into a numerical representation using attention and feedforward layers. At the same time, a similar thing happens in the decoder, where a corresponding sentence in the 2nd language (with blank “masks” instead of the words that the architecture is meant to predict) is also transformed into a numerical representation, using similar transformations. Then, the output of the encoder is passed to the decoder, where it is concatenated with the numerical representation of the L2 sentence and together they are passed through additional attention and feedforward layers. The final output is compared with the intended output, and the weights on each of the layers are updated to minimize the error between the two (Devlin et al., 2018). Once the model is trained, the encoder can be extracted from the model and be used to obtain rich, contextual text embeddings that can be used for further training (Munikar et al., 2019). The usefulness of such text embeddings stems from their ability to quantitatively describe the embedded text on meaningful dimensions the model discovered during training. A common example is the ability to use the vector in the embedding space corresponding to a given word as a direction in which one may manipulate the embedding of another word, e.g., find the representation of the word ‘man’ by subtracting the representation of ‘royal’ from that of the word ‘king’ (Ethayarajh, 2019).

In summary, the evolution of computational linguistics has led to the development of attention-based transformer models, such as GPT-3, which outperform their predecessors such as LSTMS and CCNS in processing distant words in text, with their high performance attributed not only to the vast training datasets but also to their ability to retain complex contextual information, a characteristic lacking in earlier models due to issues like vanishing gradients and computational costs.

## Word stimuli selection

We know from various studies of word processing that a multitude of factors by which we can describe words influence neural and behavioral responses in experiments using words as experimental stimuli. These include accessible features like length and frequency in the language (Hauk & Pulvermüller, 2004; Kuchinke, et al., 2007; Méndez-Bértolo et al., 2011), but also features which are not easily accessible, such as differences in semantic features and emotive content (e.g., abstractness, valence, arousal; see Citron, 2012 for a review). Here, emotional databases are an important asset, as they enable word stimuli selection for experimental manipulation and control of such factors. There however remain challenges to valid stimuli selection based on available datasets. The first stems from recent striking results that even newly discovered emotional dimensions can influence behavior with effect sizes comparable to those previously reported in the literature for valence and arousal (e.g., origin and subjective significance in experiments of Imbir et al., 2020, 2021, 2022), as well as known factors which were until recently not controlled in such studies (e.g., concreteness in experiments of Kanske & Kotz, 2007). The existence of such unaccounted-for dimensions may explain the disparity of reported results on the influence of emotional factors on behavior (such as those in the review by Citron, 2012) especially when stimuli lists are short and selected from a limited dataset. For this reason, a method of stimuli selection that would more likely produce valid stimuli lists may need to somehow reduce the influence of these unknown factors.

When constructing a manipulation of, for example, valence using emotional norms, we pick sets of negative and positive words that do not differ on some control dimensions (e.g., length, frequency, and arousal). To add an element of unsupervised control (control of unspecified factors), we propose to additionally perform semantic matching between these conditions, which involves selecting words for these groups containing words paired on their semantic features while differing in the manipulated factor. Words similar in meaning and used in similar contexts are more likely to have similar values on dimensions we did not explicitly control, such as imageability, than a random word pairing. An example of such a pairing may be the positive “peaceful”, with the negative “boring”. Both have low arousal, but even more importantly, both are approximately matched in their semantic content and connotations, while differing in the sign of the emotion attributed to the situation. This is indeed an example of a pairing found by our *stimuli descent* algorithm, which is introduced in Study 3.

## Study 1: Transformer-based norm extrapolation

We hypothesize that the use of highly contextual representations of words as input to a model trained to predict the emotional norms will be able to outperform the previous approaches in norm extrapolation. While some of the previous attempts at this task also relied on machine learning to extend affective norms, they all relied on word embeddings (e.g., Mandera et al., 2015) and thus were unable to capture the sophisticated contextual relations among distant words. Furthermore, contrary to word embeddings, the numerical representations obtained from transformers are flexible with regard to the task that they are trained on. For example, a transformer can be first trained to simply generate sentences but then retrained on a different, more specialized task such as emotion recognition in Twitter posts. This retraining will lead to slight changes in the numerical representations generated by the transformer, as the emotional information present in the relation between Twitter posts and their training labels (e.g., happy, sad etc.) will seep into the weights of the model, crystallizing specialized affective knowledge. A good example of such a model is ERNIE, which was trained on several different tasks and achieved state-of-the-art results on several NLP benchmarks at the time of publication (Yu et al., 2019). The use of models pre-trained on emotion recognition-related tasks should therefore further increase the performance of our approach. Additionally, since the transformer models have been trained for many different languages, our models will help researchers from different countries to extrapolate their norm datasets cheaply and accurately.

In the manuscript, we first provide a detailed description of the norm datasets that will be used to train the model. Afterwards, we detail the model’s architecture explaining the intuition behind choosing the right transformer module for the task. Then we describe the training regimes and present results comparing the outputs of the models to a specially designated part of the original datasets, ending with a discussion.

## Method

### Linguistic materials and data curation

In the current study, we make use of the ANEW corpus (Bradley & Lang, 1999), and a corpus collected by Warriner et al. (2013) to train and test our model. The former consists of 1030 words with accompanying rater-based metrics for valence and arousal. The latter has 13,915 words with both previous metrics and, additionally, the age of acquisition and concreteness metrics. All the metrics have been normalized to range from 0 to 1. In line with the argumentation from previous work on these corpora, we used the ANEW words for the test set, subtracting them from the training set composed of all the words present in Warriner’s database. The test set, therefore, consists of 983 words. The rest of Warriner’s corpus (12,885 words) was divided into training and validation sets at a 9-to-1 ratio Table 1 and 2.

**Table 1:** The specification details of the different models

Specifications	English	Polish	Spanish	Dutch	German	French
Transformer encoders	ERNIE 2.0 (Yu et al., 2019)	RoBERTa-Polish (Dadas, 2020)	“bert-base-spanish-wwm-cased” (Perezrojas et al., 2020)	“bert-base-dutch-cased” (de Vries et al., 2019)	"bert-base-german-uncased" (von Platen, 2021)	“french_toxicity_classifier_plus_v2**”** (Stakovskii, n.d.)
Mean number of raters	28	50	21	63	Not reported	36
Number of words	13,915	4900	1400	4299	2902	1031
Learning rate	5e-5	5e-4	5e-4	5e-4	5e-4	5e-4
Dropout	0.1	0.2	0.2	0.2	0.1	0.1

**Table 2:** Correlation results for the past extrapolation models

Study	Valence	Arousal	Dominance	Concreteness	Age of acquisition
Current Study	**0.95**	**0.76**	**0.86**	**0.95**	**0.85**
Vankrunkelsven et al., 2018	0.86	0.69	0.75	0.87	0.59
Vankrunkelsven et al., 2015	0.89	0.76	0.77	0.81	0.67
Mandera et al., 2015	0.69	0.60	0.48	0.80	0.72
Recchia and Louwerse, 2015	0.74	0.75	0.62	-	-
Bestgen and Vincze, 2012	0.71	0.56	0.60	-	-

The best results for a certain metric are in *bold*. Lack of prediction for a certain metric is signified by a *dash*

To expand our model to other languages, we make use of five additional datasets. For the Polish language, we employ a norm repository of 4900 words (Imbir, 2016). For Spanish, the dataset contains 1400 words (Redondo et al., 2007). For German we use the BAWL-R dataset with 2902 words (Võ et al., 2009). For French we use the FANCat dataset with 1031 words (Syssau et al., 2021) Finally, we employ norms for 4299 words in the Dutch language (Moors et al., 2013). Unfortunately, all of the same metrics were not available for all of the different languages. While fewer dimensions were available for Spanish and Dutch, the contrary was true for Polish, where we were able to use an eight-metric database. The availability of the metrics in each of the languages can be checked in Table 3. The metrics were normalized to the 0 to 1 range, and the datasets were split for training, validation and testing according to the 8:1:1 ratio.

**Table 3:** Correlation results of affective metrics from the three additional languages on the test set

Affective metric	English	Polish	Spanish	Dutch	German	French
Valence	0.95***	0.93***	0.89***	0.87***	0.8***	0.8***
Arousal	0.76***	0.86***	0.80***	0.80***	0.7***	0.77***
Dominance	0.86***	0.92***	-	0.75***	-	-
Concreteness	0.95***	0.95***	0.89***	-	-	-
Age of Acquisition	0.85***	0.81***	-	0.82***	-	-
Origin	-	0.86***	-	-	-	-
Significance	-	0.88***	-	-	-	-
Imageability	-	0.88***	0.86***	-	0.82***	-
Familiarity	-	-	0.71***	-	-	-

* p < 0.05** *p* < 0.01*** *p <* 0.001

Lack of the prediction of a certain metric is signified by a *dash*

### Model architecture

The proposed model maintains the same architecture across all languages and norms, with the only variation being the transformer embedding model used for each language. As transformer models are usually trained for singular languages at a time, we cannot use a model that uses all languages at the same time. Beyond issues of accuracy, this could pose troubles related to the differences in norms between different languages (Pires et al., 2019). To facilitate the use of our architecture in new languages, the selection of the embedding model is explained in Appendix 1. We only use the encoder from the transformer model as the base encoding layer for our model, and we build additional layers on top of it. The added layers consisted of a single fully connected layer with layer normalization and another layer with one number as its output. On top of that, we have applied a sigmoid activation function, which ensures that the output of our model yields a normalized value between 0 and 1. This type of regression head was added for each of the predicted metrics.

Because models of similar infrastructure can be trained for different languages given enough training data, they can be fully substituted for each other, and similar models can be trained using them. This makes the proposed architecture versatile and open to being implemented in different languages. Transformer models have already been trained in many different languages and are freely available online (Hugging Face, n.d.). Therefore, for most of the languages, the only thing needed to prepare a similar model is a dataset with affective measures.

However, choosing the right transformer for the base of the model is not as straightforward. Wherever possible, we have opted for models that either had more parameters and could therefore model language more accurately, or were pre-trained on emotion recognition tasks. However, the scope of our search was limited by both hardware constraints and model availability. Since different transformer models are pre-trained on various tasks, their performance on a specific task like ours may vary. If researchers want to train a similar model for a language that is not covered in our article, we advise them to run tests using all the different transformer models available in their language, until they find the one that rears the best predictions (see Appendix 1 for more information).

The specifications for each of the models are shown in Table 1. The hyperparameters for our machine learning model were chosen according to common practices, wherein we used a combination of domain knowledge, model complexity considerations, and computational efficiency to guide the selection, minimizing the risk of overfitting and ensuring optimal performance.

Each of the models was trained for 1000 epochs with early stopping (stopping the training before the model starts overfitting the training data). This was implemented by saving the model that had the best correlations with the validation metrics. We used the AdamW optimizer algorithm with an epsilon value of 1e-8, a weight decay of 0.3, amsgrad, and betas equal to (0.9, 0.999). Additionally, we implemented a warmup algorithm, which gradually elevated the learning rate for 600 learning steps, when it reached the maximum number, and then slowly lowered it, until the end of the training. The rest of the specifications like the learning rate and the value of the dropout can be found in Table 1 as it was language specific.

## Results and discussion

The English model’s predicted affective norms achieved the following Pearson correlations with human judgements on words from the test set: valence: *r =* 0.95, arousal: *r =* 0.76, dominance: *r =* 0.86, age of acquisition: *r =* 0.85, concreteness: *r =* 0.95, with an overall loss of 0.003. When compared to the previous methods (see Table 3), the present approach achieves the highest accuracy across all variables. This is true even compared to Vankrunkelsven et al. (2018) results after they have been adjusted for attenuation (*r =* 0.91, *r =* 0.83, and *r =* 0.85 for valence, arousal, and dominance respectively). The transformer-based model has therefore been shown to achieve higher accuracy when compared to the extrapolations reported based on LSA, and other word embedding methods (Bestgen & Vincze, 2012; Recchia and Louwerse, 2015), to methods based on human word association data (Vankrunkelsven et al., 2015), those based on simple machine learning methods (Mandera et al., 2015), as well as those combining the last two (Vankrunkelsven et al., 2018). It is worth pointing out that direct comparison was not always possible as the past models did not utilize the same high-quality validation set – the ANEW corpus. However, the improvement over those that did use it is so big that the change in the test set most probably would not change the overall conclusion.

**Fig. 1:**
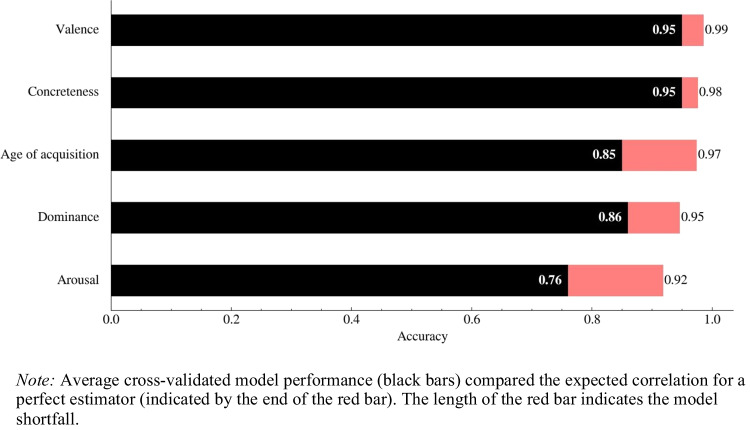
The comparison of the accuracy achieved by the model to the perfect estimator correlations. *Note:* Average cross-validated model performance (*black bars*) compared the expected correlation for a perfect estimator (indicated by the *end of the red bar*). The length of the red bar indicates the model shortfall

Given the very high observed correlations we can compare their values to the theoretically highest correlation values we can expect for the norms of the test set. The uncertainty associated with a prediction may be broken down into epistemic and aleatoric uncertainty. The former concerns the model shortfall that may be amended with better models, the latter concerns the uncertainty inherent to the studied phenomena. For norms, we may estimate the aleatoric uncertainty from the reported standard error associated with the number of raters and the variance of their judgments. With this information we can calculate the limit on prediction performance, which is reported in Fig. 1. In the next section, we discuss other sources of aleatoric uncertainty that limit extrapolation performance. Given these results, along with improvements of around Δr = 0.1 on every metric, we can safely assume that the transformer model constitutes the current state of the art in norm extrapolation. Furthermore, due to the high popularity of transformers, the current architecture can be easily adapted to different languages. This is evidenced by the results achieved on the subsets of Polish, Spanish, German, French, and Dutch words, most of them being very high (see Table 2). The ease with which the current approach can be adapted to extrapolate word norms in different languages is an improvement on the previous methods, most of which relied on human-based word associations data (Vankrunkelsven et al., 2015, 2018), which is not freely available for most languages.

Thanks to its high accuracy, the current model can be used to provide approximations of human judgements in contexts where the actual norm values do not need to be known with precision. The extrapolated norms should not be used as independent variables in linguistic studies of large corpora. As we will investigate in the next section, the predictions could be biased towards uncommon words that appear in corpora but are unlikely to be used in experimental research. In cases where precision is needed, the model can still serve as a useful heuristic tool to identify words with specific values, which can be in turn verified experimentally. For example, the model can help identify words whose specific combination of affective metrics are rare, like those with neutral valence and high arousal, to help populate specific stimuli sets, as such words are useful in studies which try to orthogonally manipulate emotional dimensions.

The possibility of using transformer-based extrapolation for the task of finding rare emotion-combination words is important, as k-nearest neighbor approaches often generalize worse in sparse regions, such as in these specific configurations of affective dimensions. If this generalization performance is confirmed it would establish a heuristic search for low-density words and extrapolation for unusual, low-frequency words as unique use cases of our method. However, we need to tackle a significant issue related to measuring extrapolation performance, highlighted by Snefjella and Blank (2020). This problem becomes known when we view norm extrapolation as a missing data problem. In short, the use of supervised learning to impute missing data, conditional on observed data is equivalent to single regression mean imputation, which is an imputation method known in the field of causal inference to produce biased estimates of accuracy via cross-validation (Van Buuren & Groothuis-Oudshoorn, 2011). What Snefjella and Blank (2020) rightly point out is that the set of words that do appear in norms databases is not random nor representative of all words in a language, creating a “missing not at random” problem in norm extrapolation. Words that are longer, less common, or more abstract all have a lower probability of appearing in norms databases, thus also the test set, resulting in accuracy bias. In Study 2 we use these insights to test how biased is the cross-validation estimate of accuracy, as well as how good our transformer-based method is at prediction generalization on different emotional dimensions. To this end, we conduct tests both with normative and new experimental data.

The results of this section, while showing promise in the ability of the model to generalize to unseen words, suggest it cannot overcome the issues with norm extrapolation to all words highlighted by Snefjella and Blank (2020). For a large number of words, the large aleatoric uncertainty in norms and the systematic bias from mean imputation largely coincide, causing an irreducible error that prohibits valid prediction with ad hoc methods. Additionally, the systematic bias in extrapolated norms can hide some complex relationships between the examined variables, which prohibits the use of extrapolated norms in corpora studies and demands researchers experimentally verify the norms in orthogonal designs.

## Study 2: Evaluating robustness in out-of-distribution prediction

The goal of this section is to test how well the transformer-based extrapolation method generalizes under selection bias and assess which words' prediction is affected by the “missing not at random” problem. Recall that prediction uncertainty may be divided into epistemic and aleatoric uncertainty. The former is associated with the quality of the model, and the latter with the variability inherent to the studied phenomena. The use of norm extrapolation should be limited to words for which the prediction error is small – first to words that have low aleatoric uncertainty (for which prediction is possible), and next to words for which the epistemic uncertainty is low, which depends on the extrapolation method. Assuming cross-validation performance applies to all words runs into discounting both the missing not at random problem and the existence of words for which the concept captured by the norm does not apply the same way as to words in the dataset. To understand the latter point, take the norms for age of acquisition. To obtain the norms, Kuperman et al. (2012) asked participants to answer at what age they thought they had learned a given word. However, more than 50% of the words were not known by all respondents, which for this measure would imply the age of acquisition was larger than the age of the participant making the norm calculated only on respondents that knew the word biased downwards. Here, therefore, we encounter the first limitation of our method, as age of acquisition norm extrapolation should not be used for large values. We conduct three tests for other metrics to assess the two sources of additional prediction error: (a) stemming from the larger aleatoric uncertainty of words unlikely to appear in the test set (b) stemming from a larger epistemic uncertainty in out-of-distribution prediction.

We start by testing whether our method produces biased results under a meaningful selection bias. Abstract and concrete emotional words are processed differently by people, mediating the effects of valence and arousal on reaction times and the neural response (Kanske & Kotz, 2007). Concreteness is thus a good candidate for a factor that may bias results if selected not at random. We test the robustness of our method to additional systematic sampling bias by predicting norms of abstract words using a model trained only on concrete words. Next, we take advantage of English concreteness norms existing for a much larger set of words than the set of words in the test and training sets and establish how accuracy decreases for words known by fewer people. Lastly, we collect additional norms for words chosen entirely at random to find a lower bound for unbiased accuracy across the entire language.

## Method

### Design and linguistic materials

To test the generalization performance of our method, which the extrapolation methods need for accurate out-of-distribution prediction, we train a model within an artificially imposed selection bias. The English corpus used to train the original English model was resampled to include only words with a concreteness value above the center of the distribution, corresponding to a prediction of 0.5 in the original model (where low values are abstract), leaving 6307 words for training. We then constructed two test sets. The first included only highly abstract words (with concreteness < 0.5; 364 words), simulating the prediction of norms outside the database. The second contained a randomly selected test set from all words, matched on word length with the former. The models were trained in the exact same way as the English model in Study 1.

The norms for abstractness were taken from the dataset of Kuperman et al. (2012), which has two important features: (a) it contains an extremely large, compared to other databases, selection of 40,000 English lemmas, (b) the authors report the proportion of participants that knew the word. In the dataset, there were 27,000 single-word lemmas. We test whether there is a drop in accuracy compared to the accuracy calculated with cross-validation on words from Warriner et al. (2013). Second, we test how the accuracy decreases when decreasing the amount of people familiar with the word. General familiarity is strongly associated with the probability of being included in normative databases, as unknown words are not only hard to obtain norms for but also are not useful for experimental studies.

For the experimental validation, a set of completely random Polish words conditional on not appearing in normative norms database was created. First we gathered a list of all words that appeared at least five times in Polish language corpora (following Kazojć, 2011) and obtained a set of 31,967 words. From this set, we have randomly drawn 200 words. The first 150 words that did not appear in the polish norms database were chosen to be rated on five dimensions – valence, arousal, dominance, imageability, origin – 30 different words per dimension. The last dimension is unique to the Polish norm dataset and refers to the origin of emotional load from either more automatic or reflective processes. The choice to rate a different set of words for each dimension will bar us from inferring which dimension suffers the most from out-of-distribution prediction, but it will give us a more accurate estimate of the performance drop-off for all dimensions (as the same words for each dimension would make the drop-off more of a function of the particular word selection).

### Participants

The validation study included 89 Polish-speaking participants (47 women, 42 men). The participants' mean age was 22.6 (SD = 4.1). The study was promoted on Facebook, specifically targeting college students, as the original ANEW Polish norms (Imbir, 2016) were rated by students from this demographic. To stimulate participation, we held a drawing for a 50 PLN reward, which participants were eligible for upon completion of the study. Ratings from 66 participants (50% male) entered into the analysis after removing participants whose answer’s reliability was smaller than 0.8.

### Procedure

The study was conducted through Qualtrics. We aimed to replicate the most relevant aspects of the procedure used in the normative study by Imbir (2016). Each participant was given two emotional dimensions to assess. Before each dimension started, participants read a detailed description of the dimensions and saw a scale taken from the normative study. Participants rated words on a nine-point and answered a yes /no “Do you know this word?” question. Participants rated 30 words for each of the two dimensions, five words were repeated to assess rater reliability. This procedure differed from the normative study in that the normative study was done with pen and paper on a list of words, whereas the words appeared individually (on different questionnaire web pages) in our online study.

## Results and discussion

We calculated accuracy metrics on out-of-distribution words (e.g., accuracy of valence prediction for highly abstract words, when the model was only shown concrete words during training), and the test set drawn from all words for the four affective metrics which were not manipulated. Below, the results are shown and compared to the accuracies of the original English model in Table 4. Again, all correlations were significant with *p* < 0.001. The result shows that the transformer-based predictions generalize completely over concreteness, only with regards to the metrics of arousal and age of acquisition, with a drop in correlation of results ranging from Δ*r* = 0.1 in the case of arousal to Δ*r* = 0.15 in the case of the age of acquisition. Valence and dominance were predicted with exactly the same accuracy in both conditions.

**Table 4:** The results of the concreteness dependent missingness robustness check on the test set

Condition	Valence	Arousal	Dominance	Age of acquisition
Original model accuracy	0.95***	0.76***	0.86***	0.85***
Random test set	0.94***	0.76***	0.86***	0.86***
Test set of abstract words	0.94***	0.67***	0.86***	0.71***

* p < 0.05** *p* < 0.01*** *p <* 0.001

The original model accuracy relates to the results presented in Study 1. The concreteness manipulation relates to the accuracies of the model trained on high concreteness words and tested on low concreteness words. The comparison dataset relates to a model trained on a random sample of original words, keeping the length of the words in each of the datasets the same as in the case of the concreteness manipulation

**Fig. 2:**
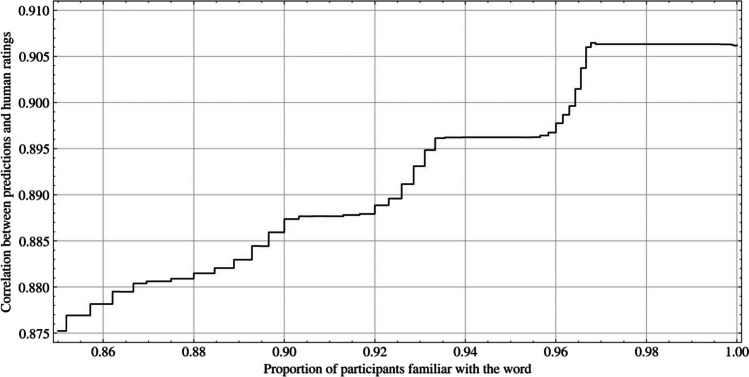
The effect of word familiarity on prediction performance for concreteness. The *y*-axis indicates the correlation of prediction and ratings estimated from a selection of words that were known by at least the proportion of participants indicated on the *x*-axis

We estimate the correlation with human judgments on a large set of concreteness norms, none of which were included in the training set. We observe that the accuracy of our model decreases slightly on out-of-distribution words and when norms of words known by fewer people are included (shown in Fig. 2, all estimated correlations were significant with *p* < 0.001). First, estimating accuracy on all 6000 words, known by all participants among words not included in the training set, yields a correlation of *r* = 0.91, slightly lower than the original test set correlation of *r* = 0.95. The correlation decreases by around 0.03 to *r* = 0.875 for all words in the concreteness norms dataset, which includes words known by at least 85% of participants.

In the experimental validation, the words were chosen at random conditional on not being included in the emotional norms database. Mean ratings were calculated as an average of the mean ratings within each gender to control for the imbalanced participant sample within each rated dimension. Descriptive statistics for all words are available in the Supplementary Materials.

Every time new norms are collected, we expect to see a larger error caused by regression to the mean, a different participant sample, rating procedure, word selection, a finite number of participants, as well as the change of emotional load of words over time (e.g., the word “pandemic”). We can quantify the change in correlation due to additional noise as the percentage change (1-r2/r1), where r1 is the original correlation and can r2 is the experimental correlation. Averaging over the five variables (see Table 5), the average drop in accuracy on out-of-distribution words equaled Δ*r* =11% (95% CI [5%, 19%]). Even for the worst value inside the confidence interval, less than 20% of the accuracy is lost on out-of-distribution words. Note that one should not compare the drop in accuracy between dimensions reported here, as each experimental accuracy was obtained on a different set of words.

**Table 5:** The results of the experimental validation test

Condition	Origin	Imageability	Dominance	Arousal	Valence
Original model accuracy	0.86***	0.88***	0.92***	0.86***	0.93***
Experimental accuracy	0.84*** [0.73, 0.92]	0.71*** [0.52, 0.82]	0.86*** [0.73, 0.93]	0.83*** [0.68, 0.92]	0.70*** [0.45, 0.89]

* p < 0.05** *p* < 0.01*** *p <* 0.001

Original model accuracy relates to the results presented in Study 1. 95% Confidence intervals are presented in square brackets. The 95% confidence interval for the original model is tighter than the precision with which correlations are reported. The “condition” column relates to the experimental condition, while the rest of the columns relate to the correlations between the respective emotional norms and their predictions

## Study 3: Stimuli descent algorithm

To demonstrate the utility of neural norm extrapolation, we propose a method leveraging the differentiability of the neural network that predicts emotional norms to select words for experimental stimuli. Here, we aim to manipulate certain emotional factors while controlling others. The algorithm, which we call *stimuli descent*, has the ability not only to control specified factors but to control other, unspecified semantic properties of words in an unsupervised way. To understand how this may be achieved, recall how word embeddings describe words on meaningful dimensions the model discovered during training. This fact is utilized, for example, in the wide use of the distance in word embedding space as a measure of semantic similarity (Kenter & de Rijke, 2015; Sitikhu et al., 2019). Thus, words that are close together tend to have similar meanings, which is the first way one can achieve control on semantic dimensions. The second way rests on the observation that the predictive models we have trained show a mapping from word embeddings to the emotional norms that is locally linear for all norms. This means that, locally, there is a single direction associated with a change in valence and this fact may be used to find a word whose embedding differs principally on this dimension, while being close to all others. By doing this, we increase the chance the matched word will be close in value on all unaccounted-for dimensions that these directions describe. This, in turn, decreases the chance that one of these outside dimensions may, for example, differentially affect reaction times in two conditions of an experiment, challenging its internal validity.

The stimuli descent algorithm takes advantage of the differentiability of our method to find semantically matched words by performing gradient descent in word embedding space with respect to the predicted emotional norm. As the position in word embedding space encodes information about how the word is used and its semantic connections, words that are close together in this space tend to have similar meanings (Stratos et al., 2015). Thus, stimuli descent moves down the function from word embeddings to norms to find the closest word that differs in this norm, allowing for the selection of semantically matched words. To do this, the algorithm at each step predicts the norms and calculates the gradient of the norm we wish to manipulate. As this gradient is the vector in word embedding space that points in the direction of the fastest increase in the predicted rating, the algorithm moves along this vector to find the closest word with the most divergent rating. https://colab.research.google.com/drive/1Cjcejg1AdDhsZWs4VioQ5tT8B496jgFZ

## Method

We wish to find semantically matched words less or more pronounced on the manipulated dimension, for words in a set. To simplify the presentation of the algorithm, we will assume our manipulated dimension is valence, and we wish to match words less positive than those from a set of positive words. Apart from the word we must specify the minimum difference in ratings we wish to achieve (denoted ${\Delta }_{min}r$ ), so that the difference (denoted $\Delta r$) between a matched word’s valence and the valence of the original word is at least as large as ${\Delta }_{min}r$. Now, we must define the loss function with respect to which we will perform gradient descent. In this instance, it will be the predicted valence rating (with either a plus or minus to increase or decrease the rating). We may also wish to control other emotional dimensions such as arousal. To this end, we may perform a kind of controlled gradient descent, where we want to remain at the same level of the controlled dimensions at each step. Thus, at each step we calculate the prediction of the controlled dimension. The gradient of this prediction shows the direction in which the prediction of controlled dimensions changes and we may use these “controlled gradients” to remove their components from the loss gradient, remaining approximately at the same level of controlled dimensions.

**Fig. 3:**
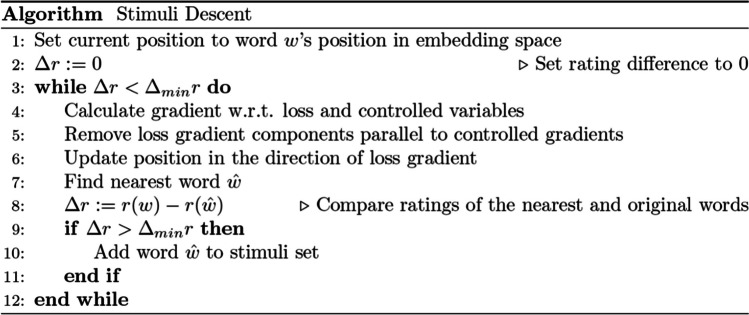
The description of the stimuli descent algorithm

Last modifications to the algorithm involve accounting for the high dimensionality of the space we are moving in. First, as words are discrete points in this high-dimensional embedding space, there may not be a word that corresponds to the place this procedure has moved us to. Thus, to select new stimuli we need to check for approximate matches – words that are close in embedding space according to some distance metric. We chose cosine similarity, a metric typically used for comparing embedding similarity. Second, this high dimensionality makes it easier to move to regions of word embedding space where there are no words. This creates the issue that in such regions the network predictions are only extrapolations, which could easily be wrong. Thus, we add to the loss function a regularization term penalizing the algorithm for stepping outside of a multivariate gaussian distribution approximating the word occurrence distribution. This term is proportional to the logarithm of the Gaussian density function and is described in more detail in Appendix 1 along with other technical details on the objective function. The complete algorithm is described in Fig. 3.

Figure 3 The stimuli descent algorithm, which finds semantically matched words via controlled gradient descent in the word embedding space

## Results and discussion

For matching words with the stimuli descent algorithm, we have trained another transformer model for English, but with just one word embedding space, instead of two (with embeddings from the BERT model "bertweet-base-emotion-analysis"; Pérez, 2021). The correlations with human judgments for this model were similar to the ones from Table 2 and evaluated to be *r* = 0.95, *r* = 0.76, *r* = 0.86, *r* = 0.85, and *r* = 0.95, respectively for valence, arousal, dominance, age of acquisition, and concreteness.

We have looked for semantically matched words for two commonly manipulated factors: valence and arousal. While looking for similar words that differ in these dimensions, we have also instituted controlled variables, such that the algorithm was either manipulating valence and controlling arousal or manipulating arousal while controlling valence and dominance. To start, the algorithm needs a word to match the next words to. For each dimension we selected approximately 250 words. Since we can choose to either decrease or increase the ratings, half of these words were high, and half were low on the dimension of interest.

To minimize the risk of bias, the selection of words was done based purely on ratings. For the valence manipulation we picked words whose valence ratings were closest to 1 standard deviation above or 1 standard deviation below the mean valence rating. Similarly, for arousal, we picked words close to 1.5 standard deviation above or below. Selected results of these analyses are presented in Table 4. All obtained results are available in the following OSF repository: https://osf.io/cug92/?view_only=6f246610bc0b43cc9e98d7c978f2f6fa Table 6.

**Table 6:** Words generated using the stimuli descent algorithm

Manipulation of valence			Manipulation of arousal		
Low	Medium	High	Low	Medium	High
**Skeleton**	Fossil	Wishbone	**Pleasant**	Splendid	Glorious
**Crass**	Cheeky	Whimsical	**Villa**	Condo	Mansion
**Unscheduled**	X	Surprising	**Hanger**	Rake	Spikes
**Pretentious**	Contemporary	Philosophical	**Patron**	Promoter	Activist
Intimidate	Exceed	**Impress**	Willing	Attentive	**Eager**
Drain	X	**Fountain**	Bike	X	**Motorcycle**
Subdue	Neutralize	**Alleviate**	Mule	Possum	**Skunk**
Insurance	Consumption	**Income**	Unintentional	Overwhelming	**Uncontrollable**

Sample results of semantically matched words. *Bold font* indicates words that were put to the algorithm, the words in the next two columns have been matched by the algorithm. ‘X’ indicates the algorithm did not find any matching words for that level of the manipulated dimension

Observing results, we see that successful matches sometimes also automatically match words also on more surface level features, such as length, or how the word sounds. One such example from the supplementary electronic material is the match “trample” and “scramble”. This is useful as such surface-level features have also been shown to influence behavior in experimental tasks (Hauk & Pulvermüller, 2004; Kuchinke, et al., 2007; Méndez-Bértolo et al., 2011). Matching stimuli with neural networks present an exciting direction for methods research that may lead to more robust experimental results with word stimuli.

Certain limitations of the method must be noted. First, certainly not every word has a word of different emotional value matching its semantic content. What this means for the method is that it can certainly fail to find matching words but will nonetheless propose candidate words. Thus, caution needs to be exercised when using the algorithm to generate stimuli to identify these cases. Some methods can be developed to identify mismatched words. First, the distance of the word at the matching stage can serve as an indicator of the appropriateness of the match. This leads to the second limitation of the validity of distance measurement in high-dimensional spaces. In higher dimensional spaces, the likelihood of finding points diminishes exponentially with the number of dimensions. Future work may try to address this limitation by picking several candidate words using the algorithm and performing the selection process with more sophisticated measures of similarity.

## General discussion

The present study has shown that a transformers-based architecture can be useful in predicting a range of affective and other word metrics. While the usefulness of transformers for such tasks has been widely recognized in machine learning (Lin et al., 2022), the full extent of the benefits that these methods can bring to the study of human behavior is an open area for research. The psychologically interesting conclusions that can be drawn from the high correlation of transformer-based norm extrapolation with human judgments can provide support to the claim that the distributional properties of words in written language hold information about how word stimuli are judged in terms of their emotionality by participants of psychological studies (Sahlgren, 2008). In the end, the numerical representations of words, which we use in the training process of our models, are related to how words interact with each other in text (through the attention mechanism concept introduced by Vaswani et al., 2017). A statistical regularity therefore exists between emotion ratings and the structure of human language. The two cognitive mechanisms that can be hypothesized to support such statistical regularity are the influence of affective properties on the way language is used, and the converse mechanism of the patterns in which language is used influencing the affective reactions, either directly or through shaping the interpretation of semantic content (for a discussion of links between distributional co-occurrence and emotional dimensions see Snefjella & Kuperman, 2016). This is easy to imagine as the emotional meaning of words is well associated with their semantic meaning (Vankrunkelsven et al., 2015). This result, however, does not extend to all words of a language, as prediction is limited to words similar to the words used in emotional words databases (Snefjella & Blank, 2020).

The possibility of inferring vast amounts of information from distributional properties finds support in the natural language processing literature, tasked with extracting meaningful semantic information from text based on the co-occurrence of words in large corpora. A common example includes the algebraic treatment of vector word-meaning representations in word embeddings, using which it is possible to find the representation of the word ‘man’ by subtracting the representation of ‘royal’ from that of the word ‘king’ (Ethayarajh, 2019). More novel methods, such as the ones used in this paper, may represent an even wider array of semantic information, which is evidenced by the impressive semantic capabilities of the most advanced transformer-based large language models (Sobieszek & Price, 2022), which for GPT-3 included translation, summarization and the execution of linguistic tasks purely from their description (Brown et al., 2020), which the introduction of GPT-4 expanded to a vast set of common sense task that seem to require some basic understanding of the world (for detailed tests, see Bubeck et al., 2023).

These developments point to an increasing role that machine learning may play in the conduct of psychological studies of language and emotions. A basic use case that the high correlations with human judgments could afford is the use of extrapolated norms for choosing experimental stimuli. While empirical verification of extrapolated norms is always advised, it does not render the extrapolation useless. Say one was designing a study with an orthogonal design that studies the influence of three emotional factors, for example, valence, arousal, and dominance, with affective word stimuli. As valence is highly correlated with dominance and has a quadratic relationship to arousal (Warriner et al., 2013), it is very rare for words to have an emotional load of positive valence, low dominance, and low arousal at the same time, but a group of such words would be required to construct such an orthogonal design. This means that not enough words may be present in the available affective norms dataset to construct such a design. The issue also arises in simpler designs when attempting to control correlated factors. The solution that precise norm extrapolation affords is to use its predictions as a heuristic tool for picking stimuli to put to human evaluation to balance the existing affective word databases. Using existing affective norms, one may predict which words are likely to have the unusual emotional load of positive valence, low dominance, and low arousal, and verify this prediction empirically. In this way, semantic norms extrapolation may be used as tools for picking experimental stimuli.

To support this application of the transformer-based norm extrapolation proposed in this paper, we developed an algorithm for the selection of stimuli. The algorithm leverages both the high correlations with human affective judgments and the semantic aspects of words learnt by the network to select words that are semantically similar, yet affectively different. The algorithm allows one to manipulate emotional factors while controlling others, but also employs the unsupervised control of word meaning that has not yet been explored in the literature on affective words. The novelty of the method stems from its leveraging of the differentiability of our extrapolation method. As the method does not use k-nearest neighbors for extrapolation we can find the gradients of all the predicted norms to find the nearest word with different affective ratings, where the distance is an auxiliary measure of semantic similarity.

An additional consideration is whether our model may be of use in the study of computational models of emotion (Marsella et al., 2010). Firstly, the norms modeled by our network are the average of the outcomes of individual emotional processes of the participants of the normative study. These population-level estimates, while useful, do not correspond to the experiences of any particular person and as such the ability to infer from norms to psychological mechanisms is severely limited. Currently the use of the model in such a context is additionally limited by the general limited understanding of how transformer networks make their predictions and as such drawing any specific scientific conclusions for cognitive science from the trained model should be discouraged.

To avoid scientifically dubious conclusions, the misuse of the model may bring, it is necessary to underscore the limitations highlighted in the robustness section, following the critique of Snefjella & Blank (2020). Our transformer-based extrapolation method, while versatile showing promise in dealing with selection bias, does not overcome the limitation posed by out-of-distribution prediction from a sample from which norms are missing not at random. To address this one should avoid using imputed norms in studies where a systematic bias on uncommon words may lead to false inferences, such studies which analyze stimuli corpora. If one wishes to select as stimuli uncommon words it is necessary to experimentally validate their norms, as there both epistemic and aleatoric uncertainties will impact the model’s performance. As discussed previously, it is generally ill-advised to use extrapolated norms of age-of-acquisition and any norm of words known by a fraction of people. The conclusion of the experimental validation is that one should conservatively expect at least a 10% drop in accuracy for out-of-distribution words. Consequently, while the model may be a valuable tool, its applications should always take note of the fact it does not overcome the issues of missing data imputation highlighted by Snefjella & Blank (2020). The same issues apply to all the norm extrapolation methods reported previously as well (Snefjella & Blank, 2020).

Taking advantage of the advances in machine learning is important for the broader scientific community, especially considering the asymmetry in the availability of computational resources. Guided by this thought, we have chosen not only to share all our code and models, but to implement the methods from the paper in an online notebook that allows for their use with a simple graphical interface. In this way, the methods can be used without the need for coding or access to high-end GPUs. We hope that this will maximize the benefits that the methods can bring researchers in the psychology of emotion.

### Supplementary Information

Below is the link to the electronic supplementary material.

Supplementary file1 (PDF 123 KB)
