# Supplementary material for: Extrapolation of affective norms using transformer-based neural networks and its application to experimental stimuli selection
Source: Behav Res Methods. 2023 Sep 25;56(5):4716–31. doi: 10.3758/s13428-023-02212-3 (PMC11289359; doi:10.3758/s13428-023-02212-3)
Supplement: Supplementary file 1 — Supplementary file1 (PDF 123 KB) [file 13428_2023_2212_MOESM1_ESM.pdf]

Appendix: Implementation details

1 Embedding model selection

For each language, the selection of the embedding model followed a simple two step procedure:

First, candidate models were sourced from https://huggingface.com by searching for BERT and RoBERTa tranformer models for each language. If available, the following models were chosen: "bert-base" for BERT; "roberta-base-v2" for RoBERTa. Additionally, if a version of each model fine-tuned for sentiment analysis was available this model was preferred to the base model (e.g. the fine-tuned French BERT model "french_toxicity_classifier_plus_v2"). We also sourced the ERNIE 2.0 model, which at the time of writing was available only in English.

In the second stage, two models were trained for each language (three for English) with the same set of hyperparameters, either with the BERT or RoBERTa encoder model. Performance was measured on the validation set. We observed neither model lead to better performance for all languages, so for each language the embedding model with the higher correlation with human judgments were chosen. Two hyperparameters were optimised, the dropout rate of either 0.1 or 0.2 and the learning rate of either $5e-4$ or $5e-5$. The models were then retrained for the full 1000 epochs and evaluated on the test set.

2 Stimuli descent objective function

The stimuli descent algorithm performs gradient descent in the word embedding space W w.r.t. an objective function $\mathcal{L}$. Starting from an initial position x, at every step we wish to move in the direction of increasing or decreasing one of the predicted emotional dimensions (labelled r). As noted in the manuscript, this procedure runs the risk of moving into a region of W space, where there are no words.

Regularization To combat this, we introduce a regularization term into the objective function of the algorithm that penalises steps outside of the word distribution. First, we approximate the density of words in W with a multivariate Gaussian distribution $\mathcal{N}(\mu, \Sigma)$. We may use the probability density function (PDF) of this approximate distribution to specify whether a step moves us out of the distribution. Specifically, we take the logarithm of the PDF to construct a regularization term, as its gradient points in the direction of an increase in word density, and its length increases as we move more and more outside of the distribution.

$$\ln \mathrm{pdf}(x) = \ln[\det(2\pi\Sigma)^{-\frac{1}{2}}] - \frac{1}{2}(\mathbf{x}-\mu)^{\mathsf{T}}\Sigma^{-1}(\mathbf{x}-\mu) \tag{1}$$

Where μ is the vector of means and Σ the covariance matrix, both estimated based on embeddings of a large set of English words. To simplify calculations, the regularization term $\mathcal{L}_g$ omits constants and the slope:

$$\mathcal{L}_g(x) = -(\mathbf{x}-\mu)^{\mathsf{T}}\Sigma^{-1}(\mathbf{x}-\mu) \tag{2}$$

Adaptive hyperparameter There is no need to correct the gradient in the direction of increasing density at every step, instead we introduce a hyperparameter λ that controls the amount of correction based on whether the previous step was in the direction of decreasing or increasing density. This is calculated as the difference in the regularization loss $\mathcal{L}_g$ at the previous step x_{t-1} and this step x_t. A further hyperparameter a controls the size of the adaptive hyperparameter:

$$\lambda_t = a \cdot (\mathcal{L}_g(x_{t-1}) - \mathcal{L}_g(x_{t-1})) \tag{3}$$

Objective function The full objective function is based on the direction of manipulation $d \in \{-1, 1\}$, the manipulated dimension r and the position $x \in W$, which starts at the position of the initial word.

$$\mathcal{L} = d \cdot r + \lambda \cdot \mathcal{L}_g(x) \tag{4}$$
